# The role of PANDER and its interplay with IL-6 in the regulation of GLP-1 secretion

**DOI:** 10.1530/EC-23-0548

**Published:** 2024-10-04

**Authors:** Zeting Li, Ling Pei, Huangmeng Xiao, Nan Chen, Fenghua Lai, Shufang Yue, Changliu Xu, Yanbing Li, Haipeng Xiao, Xiaopei Cao

**Affiliations:** 1 Department of Endocrinology, Sun Yat-sen University First Affiliated Hospital, Guangzhou, China

**Keywords:** interleukin-6, glucose-like peptide-1, pancreatic-derived factor, family with sequence similarity 3b, gestational diabetes mellitus

## Abstract

Glucose-like peptide-1 (GLP-1) is a vital hormone in the intestines that regulates glucose metabolism. Although pancreatic-derived factor (PANDER) overexpression is known to suppress GLP-1, the underlying mechanisms are unclear. Our study aims to uncover how PANDER influences GLP-1 synthesis and secretion. We established a PANDER overexpression model in STC-1 intestinal cells, confirming its inhibitory effect on GLP-1 secretion. This effect was reversed in PANDER-knockout cells. Additionally, a negative correlation between PANDER and GLP-1 was observed in patients with a history of gestational diabetes. Subsequently, through whole transcriptome gene sequencing in PANDER-overexpressed STC-1 cells, we discovered that the activation of IL-6 and its related STAT3 signaling pathway was significantly inhibited, and this finding was validated by Western blotting and quantitative reverse transcription PCR. Finally, rescue experiments confirmed that the IL-6-related STAT3/Akt/GSK3β/β-catenin signaling pathway mediates the negative regulatory effect of PANDER on GLP-1. Taken together, our data identify IL-6 as a bridge connecting PANDER and GLP-1 in the STC-1 cells, demonstrating potential therapeutic targets for diabetes treatment by targeting the PANDER–IL-6–GLP-1 axis.

## Introduction

Glucagon-like peptide-1 (GLP-1) is an incretin hormone that helps regulate postprandial blood glucose levels through various mechanisms. It is primarily expressed and secreted by intestinal L cells and encoded by the glucagon gene (GCG) (1, 2). GLP-1 plays a critical role in promoting the survival of pancreatic β cells, stimulating insulin secretion in a glucose-dependent manner, inhibiting glucagon secretion from pancreatic α cells, delaying gastric emptying, and enhancing satiety (3, 4). Clinical evidence has shown a significant decrease in GLP-1 levels in patients with impaired glucose metabolism, emphasizing the importance of the gut–pancreas hormone axis in diabetes pathogenesis (3). However, the molecular mechanisms responsible for the reduced secretion of GLP-1 in patients with type 2 diabetes mellitus (T2DM) remain unknown.

Pancreatic-derived factor (PANDER), also known as family with sequence similarity 3 B (FAM3B) (5, 6), is a cytokine mainly secreted from pancreatic cells. PANDER has been shown to influence glycolipid metabolism by inducing apoptosis in pancreatic β cells, inhibiting insulin secretion (6, 7, 8), promoting hepatic glucose production, and stimulating lipogenesis (9, 10). Recent studies have reported elevated plasma PANDER levels in individuals with T2DM (11, 12, 13), metabolic syndrome (MS) (14), and gestational diabetes mellitus (GDM) (15). Previous experiments have demonstrated that exogenous PANDER treatment of intestinal L cells or lentivirus-mediated overexpression of PANDER in mice leads to a decrease in GLP-1 secretion (16). These findings suggest that PANDER plays a role in glucose metabolism and could potentially be a target for managing hyperglycemia. However, the exact regulatory mechanism of PANDER on GLP-1 remains unclear.

The impact of PANDER on GLP-1 secretion has not been fully established, and the specific mechanisms involved remain unclear. Therefore, the objectives of this study are to validate the correlation between PANDER and GLP-1 in clinical research, identify target genes influenced by PANDER, and elucidate the precise mechanisms underlying its impact on GLP-1 secretion.

## Research design and methods

### Cell culture

STC-1 cells, obtained from the American Type Culture Collection, were cultured in low-glucose (1 g/L) DMEM containing 10% fetal bovine serum (FBS) and 1% penicillin and streptomycin (PS) at 37°C with 5% CO2 in a 95% relative humidity environment. The medium was exchanged every 3 days, and the cells were trypsinized and reseeded at a 1:3 dilution when they reached 70–80%. For quantitative reverse transcription PCR (qPCR), Western blot, ELISA, and immunofluorescence tests, STC-1 cells were plated in six-well culture plates and allowed to reach 80–90% confluency.

### Construction of PANDER overexpress STC-1 cell line

The mouse PANDER gene sequence (Gene ID: 52793) was obtained from the National Library of Medicine’s gene bank. To generate cell lines that overexpress PANDER, a PANDER adenovirus was procured from Gene Pharma (Shanghai, China), and its sequence is presented in Supplementary Table 1 (see section on supplementary materials given at the end of this article). Additionally, to further investigate the underlying mechanism, the PANDER gene was cloned into the GV367 vector (Gene Chem, Shanghai, China), which contains the Ubi-MCS-SV40-EGFP-IRES-puromycin component sequence, via AgeI/NheI restriction sites. Lentivirus was produced by transfecting 293T cells with the PANDER plasmid utilizing the manufacturer’s protocol. Subsequently, STC-1 cells were then transfected with lentiviral supernatants containing the PANDER gene and subsequently screened with puromycin to identify the desired PANDER-overexpressing cell lines.

### Construction of PANDER knockdown STC-1 cell line

To establish a stable PANDER-knockdown STC-1 cell line, we utilized CRISPR/Cas9 methods (17, 18). LentiCRISPR V2 vectors were obtained courtesy of Professor Weidong Ji (The First Affiliated Hospital of Sun Yat-sen University). The sgRNA targeting PANDER was 5′-CCTTCGATCTGGATCTCCGC-3′ and was inserted into the LentiCRISPR V2 vector via BsmBI-v2 restriction endonuclease digestion (New England Biolabs, USA). Plasmid transfection into 293T cells was performed using Lipofectamine 3000 according to the manufacturer’s instructions (Invitrogen). The resulting lentiviruses were utilized to transduce STC-1 cells with 8 μg/mL polybrene (Sigma). After 48 h of transduction, cells were screened for PANDER knockdown by selecting with 20 μg/mL puromycin for 3 days, resulting in stable PANDER-knockdown STC-1 cell lines.

### Construction of PANDER and IL-6 double knockdown STC-1 cell line

The STC-1 cell line with dual knockdown of PANDER and IL-6 was constructed based on the STC-1 cell line with PANDER knockdown mentioned above, using the same method. The sgRNA targeting IL-6 was 5′-ACTGATGCTGGTGACAACCAC-3′.

### Genome-wide transcriptome profiling

Total RNA was extracted from STC-1 cells with PANDER overexpression and control STC-1 cells, and the quantification was performed. For single-end sequencing, cDNA libraries were constructed utilizing the SYBR Premix Ex Taq II kit (Takara Bio, cat. # RR036A) following the manufacturer’s instructions. The resulting cDNA libraries underwent Proton Sequencing process using commercially available protocols and were sequenced by NovelBio Corp. Laboratory, Shanghai. Supplementary Table 2 provides the relevant data. We evaluated the gene expression levels of transcripts obtained through transcriptome sequencing. Fragments were subjected to differential screening using the EB-Seq algorithm, and mRNAs were selected based on Log2 fold change (FC) values greater than 0.585 or less than −0.585 and false discovery rate (FDR) less than 0.05. Significant differentially expressed genes were further analyzed and visualized through heatmap generation. Gene Ontology (GO) Analysis was used to perform significant functional analysis of these significant differentially expressed genes using Fisher’s method. GO terms with a *P*-value < 0.01 were selected to construct a GOTree depicting interrelationships among different functional categories. Pathway analysis was performed using Fisher’s exact test to identify significantly enriched pathways for the differentially expressed genes.

### Quantitative reverse transcription PCR analysis

Total RNA from cells was isolated and extracted using TRIzol reagent (Life Technologies). cDNA was synthesized using the SYBR Premix Ex Taq II kit (Takara Bio, cat. # RR036A) and primer pair sequences listed in Supplementary Table 1. Amplification of the target RNA was performed using SYBR Green PCR Master Mix (Takara Bio, cat. # RR420A) and the CFX96 Real-time PCR System (Bio-Rad), with 18S used as the internal control. The expression level of the target gene was determined using the 2^−ΔΔCt^ method.

### Western blot

Western blotting was performed according to previously established experimental methods (16). Cell lysates were prepared using Radio Immunoprecipitation Assay (RIPA) buffer (1×) with phosphatase inhibitor and protease inhibitor (100:1:1; Cell Signaling Technology). Primary antibodies, including PANDER antibody (1:1000, ab103154; Abcam), β-catenin antibody (1:1000, ab32572; Abcam), STAT3 (1:1000, #9139; Cell Signaling Technology), p-STAT3 (1:1000, #9134S; Cell Signaling Technology), AKT (1:1000, #9272S; Cell Signaling Technology), p-AKT (1:1000, #4060S; Cell Signaling Technology), GSK-3β (1:1000, #12456; Cell Signaling Technology), p-GSK-3β (1:1000, #5558; Cell Signaling Technology), α-Tubulin antibody (1:1000, #3873; Cell Signaling Technology), and GAPDH (1:1000, #51332; Cell Signaling Technology), were used for the following treatment. Secondary antibodies used included anti-mouse (1:2000, ab6789; Abcam) or anti-rabbit (1:2000, 7074S; Cell Signaling Technology).

### Immunofluorescence

Cells were fixed in 4% paraformaldehyde in PBS for 20 min and then blocked using 1% bovine serum albumin for 1 h at room temperature. Primary GLP-1 antibodies (1:100, ab22625, Abcam) were diluted in the blocking solution, and the cells were incubated with the antibodies at room temperature for 2 h. Subsequently, Alexa Fluor 488 goat anti-mouse IgG (H&L) (1:1000, ab150113, Abcam) was added to the cells and incubated for 1 h at room temperature. Nuclear staining was performed using 1× DAPI (Solarbio, S2110), and the images were captured using a confocal microscope (LSM880, Carl Zeiss).

### ELISA

PANDER and total GLP-1 levels in both the serum and supernatant were measured using commercially available ultrasensitive PANDER ELISA kit (Cloud-Clone, SEL507Hu, China) and GLP-1 ELISA kit (Cloud-Clone, CEA804Mi, China), respectively, following the manufacturer’s instructions. IL-6 levels in the supernatant were measured using an IL-6 ELISA kit (Cloud-Clone, SEA079Mu, China), also following the manufacturer’s instructions.

### Clinical study

Participants were recruited from a GDM cohort that was started in 2015 (19). A total of 120 women with a GDM history and who completed a follow-up visit 6 months postpartum were enrolled. The Ethics Committee of The First Affiliated Hospital of Sun Yat-sen University approved this study (Application ID [2015]101). The related clinical study number is ChiCTR-IOR-15007181 (20).

GDM was diagnosed according to the criteria of the International Association of Diabetes and Pregnancy Study Groups (21), where any serum glucose value equal to or exceeding the appropriate threshold values during the oral glucose tolerance test (OGTT) performed between 24 and 28 weeks of gestation was considered diagnostic. The threshold values were as follows: fasting blood glucose (FBG) of 5.1 mmol/L, 1-hour postprandial glucose (PG) of 10.0 mmol/L, and 2-hour PG of 8.5 mmol/L. T2DM and IGT were diagnosed according to World Health Organization 2013 (22) criteria. A 75 g OGTT was conducted at the follow-up visit. Demographic and metabolic parameters, as well as blood samples for PANDER, GLP-1, IL-6, and insulin detection, were collected. Blood samples were centrifuged at 1000 ***g*** for 10 min at 4°C. Subsequently, 1 mL aliquots of serum and plasma aliquots were immediately frozen at −80°C. PANDER and total GLP-1 levels were measured by ELISA as mentioned above.

### Statistics

Data analysis was performed using GraphPad Prism software (version 8; GraphPad) and SPSS version 25.0. Normally distributed variables are presented as means ± s.d., non-normally distributed variables as median (interquartile range, IQR), and categorical variables as frequency (percentage). Differences between the two groups were compared using a two-tailed unpaired Student’s *t*-test, with a *P*-value of <0.05 considered statistically significant. One-way ANOVA test, Mann–Whitney *U* test, and Pearson correlation analysis were used to evaluate the relationship between different parameters.

## Results

### Inhibiting effects of PANDER on GLP-1 synthesis and secretion in STC-1 cells

**Figure 1:**
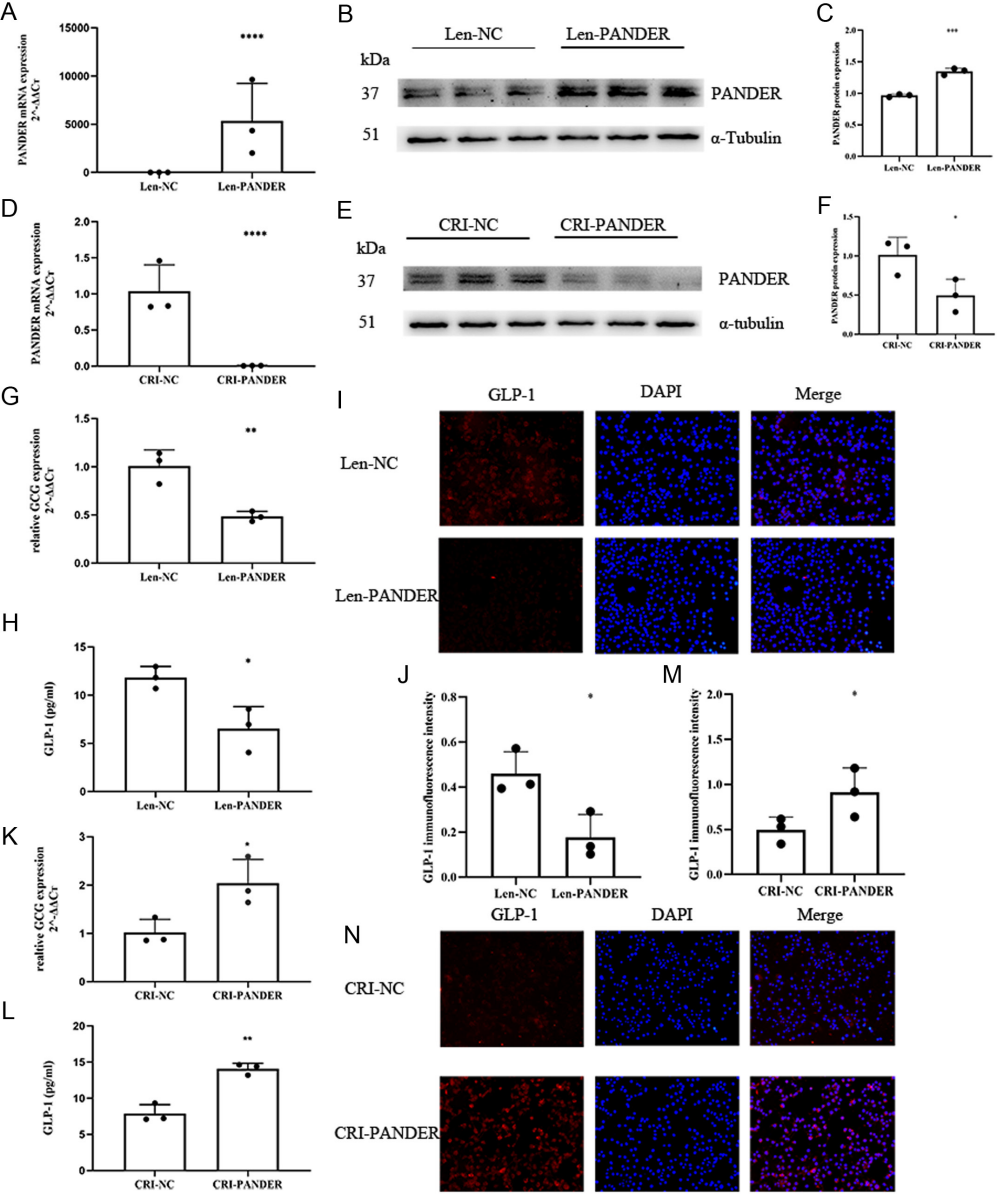
The inhibitory effect of PANDER on GLP-1 in STC-1 cells. Construction of PANDER-overexpressing STC-1 cells using the lentiviral overexpression method, validated by qPCR (A) and Western blot (B, C). Construction of PANDER-knockdown STC-1 cells using the CRISPR–Cas9 system, validated by qPCR (D) and Western blot (E, F). GCG gene expression (G), supernatant GLP-1 level (H), and GLP-1 protein expression (I, J) in the PANDER-overexpressing STC-1 cells. GCG gene expression (K), supernatant GLP-1 level (L), and GLP-1 protein expression (M, N) in the PANDER-knockdown STC-1 cells. The scale of the immunofluorescence image is 1 cm : 20 μm. * *P* < 0.05, ** *P* < 0.01, *** *P* < 0.001, **** *P* < 0.001.

To investigate the impact of PANDER on GLP-1 secretion, STC-1 cells were cultured. PANDER-overexpressing STC-1 cells were constructed using a lentivirus overexpression system, and PANDER-knockdown STC-1 cells were constructed using the CRISPR/CAS9 system. The successful overexpression and knockdown of PANDER were confirmed by qPCR and Western blot analyses (Fig. 1A, B, C, D, E and F). GCG gene expression, the GLP-1 protein in the cells, and the supernatant GLP-1 level were significantly reduced in the PANDER overexpression cells (Fig. 1G, H, I, and J), while these measures were significantly increased in the PANDER-knockdown cells (Fig. 1K, L, M and N).

### Correlation between PANDER and GLP-1 in women with GDM history

**Figure 2:**
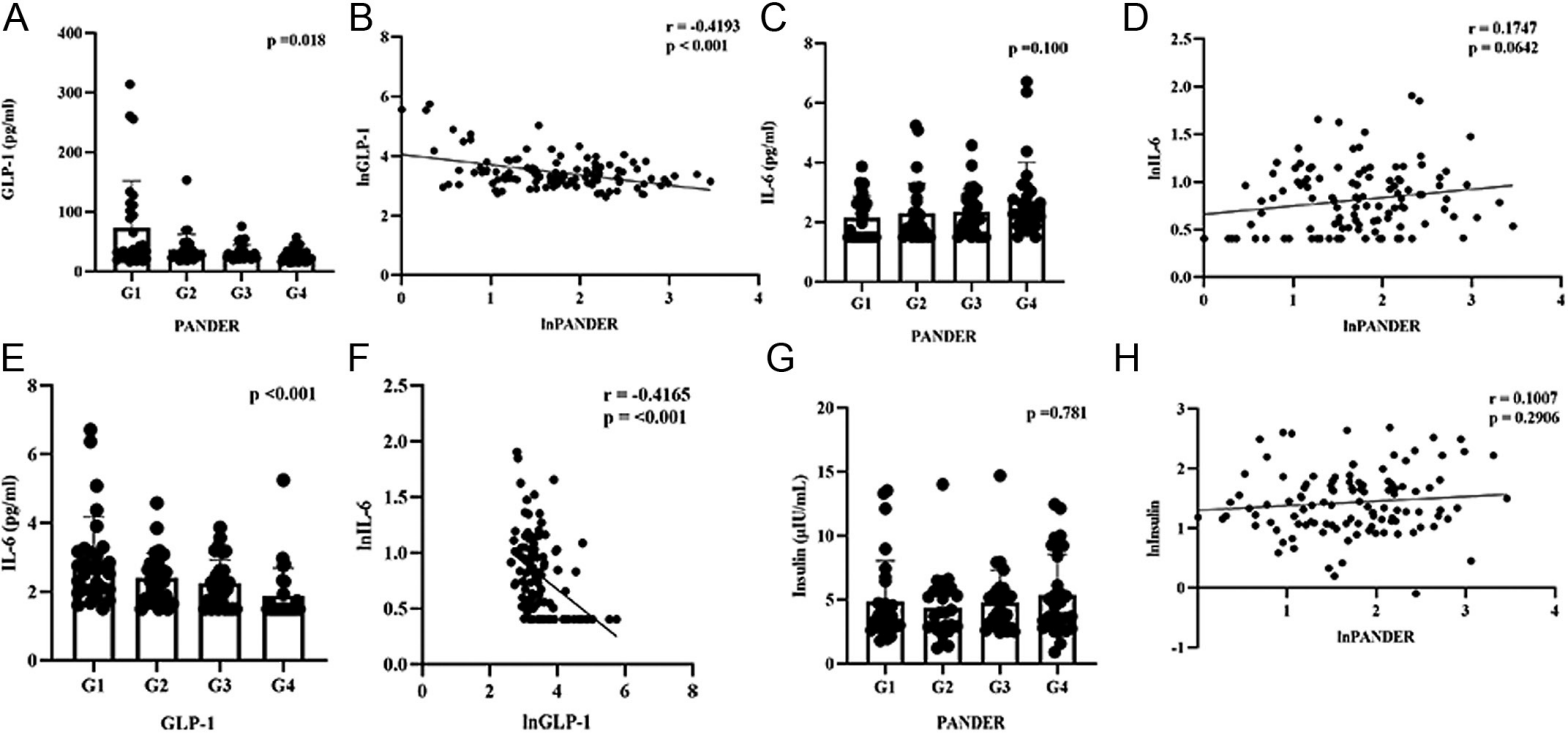
Relation of PANDER, GLP-1, IL-6, and insulin of women with GDM history. GLP-1 levels (A), IL-6 levels (C), and insulin levels (G) of GDM patients between the four groups after being divided into four groups based on the quartile value of PANDER; Pearson correlation of PANDER with GLP-1 (B), IL-6 (D), and insulin (H) of women with GDM history; GLP-1 and IL-6 levels of GDM patients between the four groups after being divided into four groups based on the quartile value of GLP-1 (E); Pearson correlation of IL-6 with GLP-1 of women with GDM history (F). G1, 0–25th value; G2, 25–50th value; G3, 50–75th value; G4, 75–100th.

One hundred twenty women with a history of GDM were recruited. They were divided into four groups based on the quartile of PANDER values at the postpartum visit. There were no significant differences among the groups in age, IL-6, fasting insulin, body mass index (BMI), systolic blood pressure (SBP), diastolic blood pressure (DBP), fasting blood glucose (FBG), or postprandial glucose (PG), as presented in Table 1. Patients with higher PANDER levels had significantly lower GLP-1 levels (*P* = 0.018, Fig. 2A). Pearson correlation analysis showed a negative correlation between postpartum PANDER and GLP-1 levels (*r* value = −0.4193, *P* < 0.001) (Fig. 2B), which is consistent with our cell experiments’ findings, suggesting a negative correlation between elevated PANDER levels and reduced GLP-1 secretion. However, Pearson correlation analysis also showed a negative correlation between postpartum IL-6 and GLP-1 levels (*r* value = −0.4165, *P* < 0.001) (Fig. 2E and F). Additionally, there was a positive correlation between IL-6 and PANDER, with a difference close to statistical significance (Fig. 2C and D), while there was no significant correlation between insulin and PANDER (Fig. 2G and H).

**Table 1:** Postpartum general characteristics of GDM patients.

	Total (*n* = 120)	G1 (*n* = 30)	G2 (*n* = 30)	G3 (*n* = 30)	G4 (*n* = 30)	*P* value
Age (year)	33.66 ± 6.16	34.50 ± 4.09	34.97 ± 4.69	32.67 ± 7.80	32.50 ± 7.17	0.293
6 months postpartum						
PANDER (ng/mL)	5.52 (3.21, 8.99)	2.16 (1.42, 2.80)	4.45 (3.63, 4.80)	6.90 (6.00, 8.15)	12.01 (10.17, 15.67)	<0.001
GLP-1 (pg/mL)	27.90 (22.79, 40.09)	34.14 (24.21, 104.21)	28.50 (24.80, 39.40)	27.41 (23.39, 35.42)	25.39 (18.27, 32.78)	0.018
IL-6 (pg/mL)	2.12 (1.56, 2.78)	1.85 (1.50, 2.72)	1.90 (1.50, 2.72)	2.19 (1.71, 2.81)	2.38 (1.89, 3.04)	0.100
Insulin (μIU/mL)	3.95 (2.92, 5.70)	3.73 (2.90, 5.16)	4.00 (2.86, 5.69)	4.25 (3.11, 5.65)	4.14 (3.00, 8.61)	0.718
BMI (kg/m^2^)	21.99 ± 2.63	20.03 ± 2.35	21.06 ± 2.68	21.99 ± 2.54	22.80 ± 2.80	0.130
SBP (mmHg)	105.32 ± 11.85	109.32 ± 15.57	105.20 ± 9.21	104.04 ± 12.69	103.09 ± 8.72	0.308
DBP (mmHg)	70.45 ± 8.85	71.73 ± 10.33	70.96 ± 8.42	68.38 ± 9.25	71.00 ± 7.40	0.570
FBG (mmol/L)	4.67 ± 0.46	4.68 ± 0.50	4.59 ± 0.41	4.68 ± 0.40	4.75 ± 0.53	0.646
30-min PG (mmol/L)	9.36 ± 1.41	9.37 ± 0.93	9.65 ± 1.71	9.23 ± 1.42	9.22 ± 1.52	0.620
2-h PG (mmol/L)	8.32 ± 2.12	8.16 ± 2.09	8.40 ± 2.64	8.78 ± 1.70	7.93 ± 2.00	0.463

BMI, body mass index; DBP, diastolic blood pressure; FBG, fasting blood glucose; GDM, gestational diabetes mellitus; GLP-1, glucagon-like peptide-1; G1, 0–25th PANDER value; G2, 25–50th PANDER value; G3, 50–75th PANDER value; G4, 75–100th PANDER value; PG, postprandial glucose; PANDER, pancreatic-derived factor; SBP, systolic blood pressure.

### Results of genome-wide transcriptome profiling

To identify the target genes regulated by PANDER in STC-1 cells, we performed RNA transcriptome sequencing in PANDER overexpression STC-1 cells. A total of 12,619 mRNAs were detected (Supplementary Table 2), and we used the EBSeq algorithm to filter the results. Genes with a Log2 FC > 0.585 or <−0.585 and an FDR < 0.05 were considered significantly differentially expressed. The data analysis workflow is illustrated in Supplementary Figure 1A. Compared to the control cells, we observed 34 significantly differentially expressed mRNAs in the ADV-PANDER cells, with 24 being significantly down-regulated and ten being significantly up-regulated, as shown in Supplementary Figure 1D. GO analysis revealed that the genes with expression changes were mainly involved in inflammation and immune responses (Supplementary Figure 1B). KEGG pathway analysis demonstrated significant changes in JAK–STAT signaling pathways, apoptosis-related signaling pathways, and TNF-α pathways, among others (Supplementary Figure 1C). JAK–STAT signaling pathways, NF-kappa B pathways, and apoptosis-related signaling pathways were the core pathways enriched (Supplementary Figure 1E). A qPCR was performed to confirm the different expression of IL-6 (Supplementary Figure 1F).

### IL-6 mediated the effects of PANDER on GLP-1

**Figure 3:**
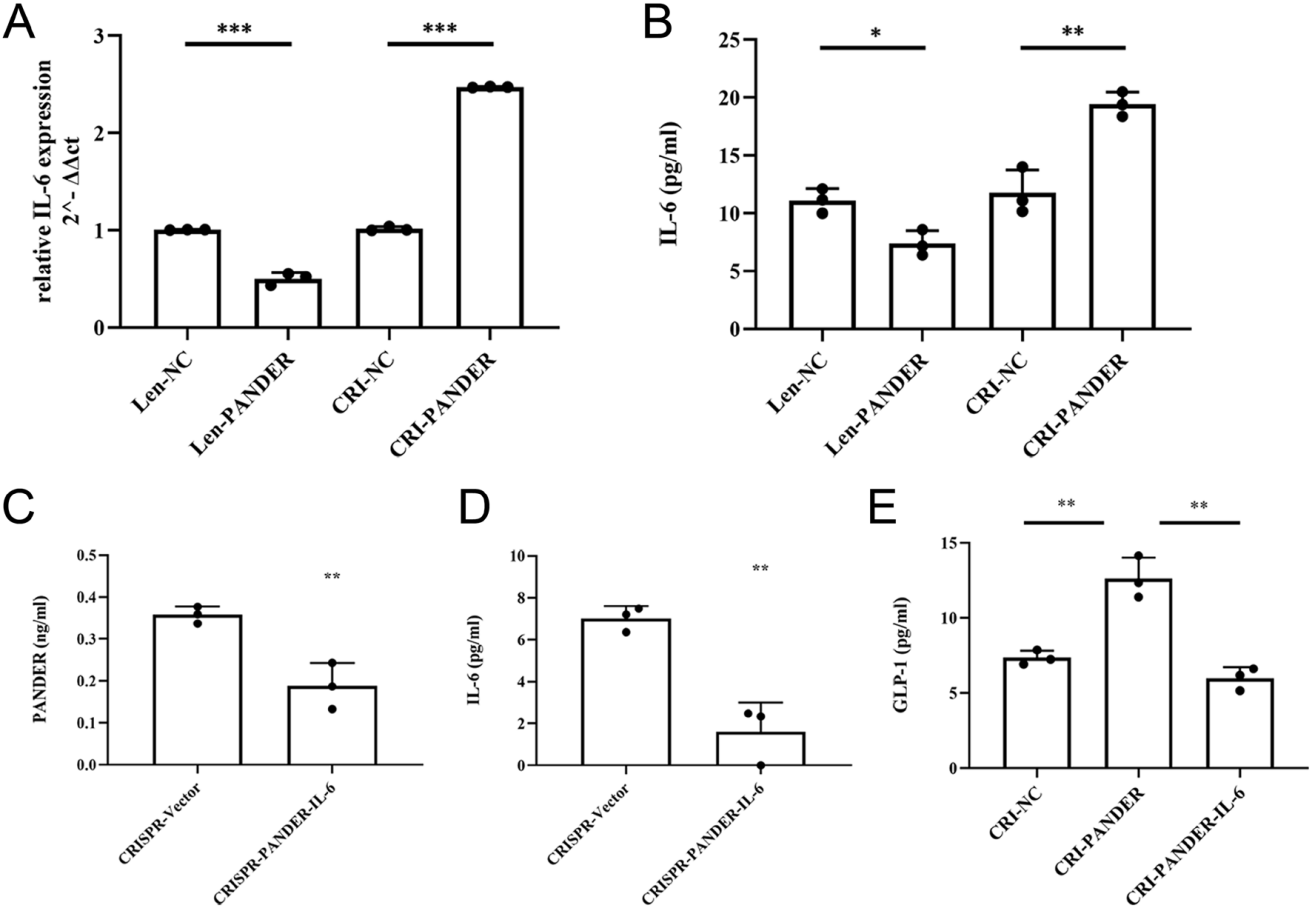
Relation between PANDER and IL-6 in GLP-1 secretion of STC-1 cells. Construction of PANDER-IL-6 double-knockdown STC-1 cells using the CRISPR–Cas9 system, validated by ELISA (A, B). Validation of PANDER-IL-6 double-knockdown in STC-1 cells was established by ELISA (C, D). After PANDER-IL-6 double-knockdown, STC-1 cells exhibited a decrease in GLP-1 secretion versus the PANDER knockdown group (ELISA, E). *P < 0.05, **P < 0.01, ***P < 0.001.

From the genome data and literature evidence (23, 24), we extracted IL-6 as a target for PANDER in regulating GLP-1 secretion. IL-6 expression and secretion were measured in PANDER-overexpressing STC-1 cells and PANDER-knockdown STC-1 cells. Our results indicated a decrease in the expression of IL-6 in the PANDER overexpression cell and an increase in the PANDER knockdown cell (Fig. 3A and B), while knockdown of IL-6 in the PANDER knockdown cell reversed the increased GLP-1 expression and secretion (Fig. 3C, D and E). These results indicated that IL-6 is a mediator in the regulatory effect of PANDER on GLP-1 secretion.

### PANDER inhibit GLP-1 secretion through IL-6/JAK/STAT3/PI3K/Akt pathway

**Figure 4:**
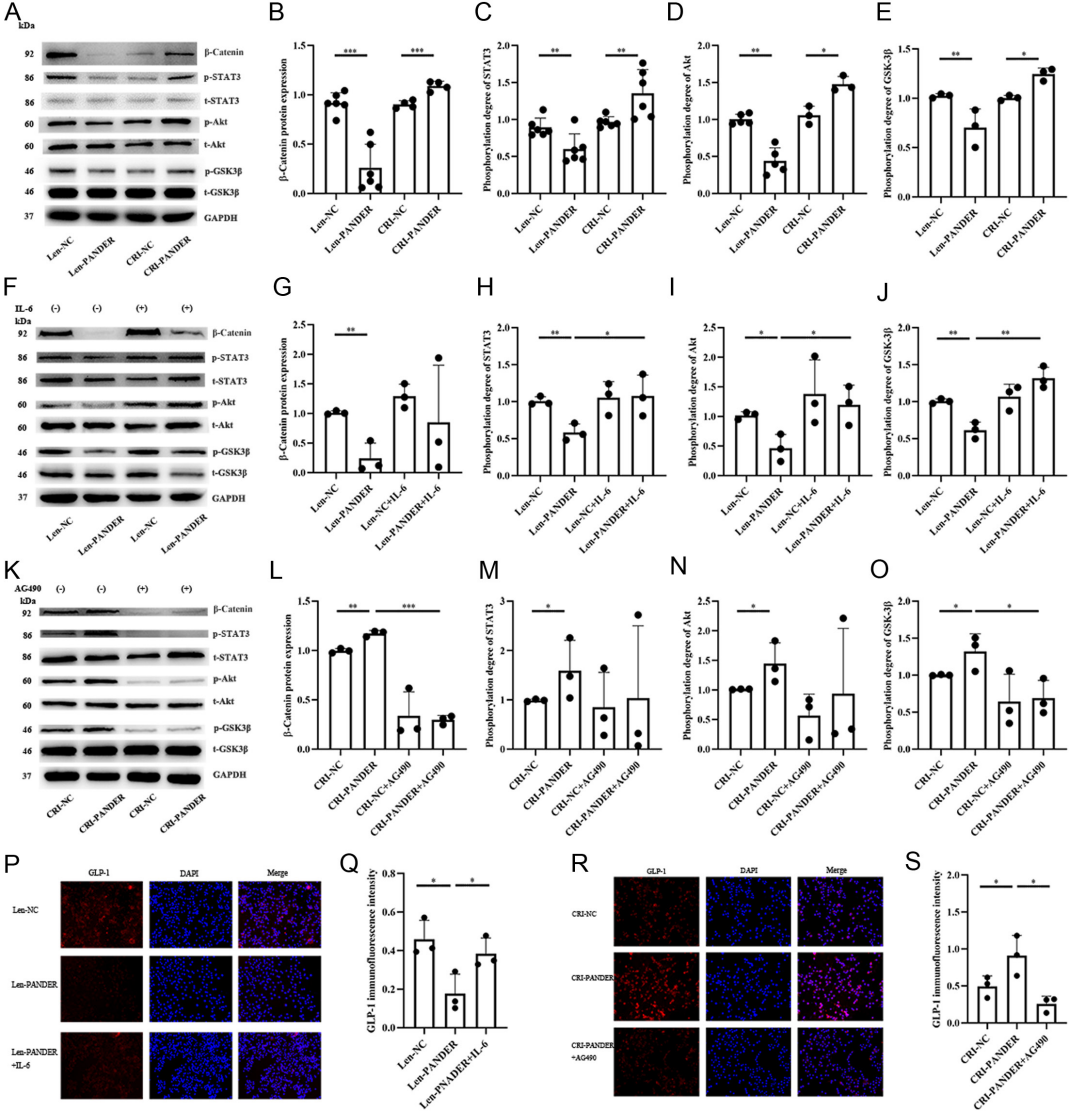
The mediated effect of IL-6 and the STAT3/Akt/GSK3β/β-catenin pathway in the inhibiting effect of PANDER on GLP-1. After PANDER overexpression, STC-1 cells exhibited a decrease in the phosphorylation of STAT3/Akt/GSK3β, and the expression of β-catenin (Western blot, A–E). The exogenous addition of IL-6 reversed the activation of the STAT3/Akt/GSK3β/β-catenin pathway (Western blot, F–J) and restored the expression of GLP-1 protein in the PANDER-overexpressing group (immunofluorescence, P, Q). In contrast, after PANDER knockdown, STC-1 cells exhibited an increase in the phosphorylation of STAT3/Akt/GSK3β, and the expression of β-catenin (Western blot, K–O). The exogenous addition of the STAT3-specific inhibitor AG490 inhibited the activation of the STAT3/Akt/GSK3β/β-catenin pathway (Western blot, K–O) and the expression of GLP-1 protein in the PANDER-knockdown group (immunofluorescence, R, S). The scale of the immunofluorescence image is 1 cm : 20μ m. *P < 0.05, **P < 0.01, ***P < 0.001, ****P < 0.001.

From our genome data and previous reports, the JAK/STAT3/PI3K/Akt signaling pathway may be involved in the IL-6 stimulation of GLP-1 secretion in intestinal L cells (3). Thus, we assessed the phosphorylation levels of STAT3/Akt/GSK3β and the expression of β-catenin in STC-1 cells. Our results have shown that overexpression of PANDER inhibited the phosphorylation of STAT3/Akt/GSK3β and β-catenin expression, while knockdown of PANDER had the opposite effect (Fig. 4A, B, C, D and E). Rescue experiments with exogenous IL-6 incubated PANDER-overexpressing STC-1 cells restored the synthesis of GLP-1 and activation of the STAT3/Akt/GSK3β/β-catenin signaling pathway (Fig. 4F, G, H, I,J,P and Q). Furthermore, when treated with AG490, a phosphorylation inhibitor of STAT3, PANDER knockdown STC-1 cells reversed the promoting effect of PANDER knockdown on GLP-1 synthesis (Fig. 4R and S) and inhibited the activation of the STAT3/Akt/GSK3β/β-catenin signaling pathway (Fig. 4K, L, M, N and O). These rescue experiments provide further evidence that PANDER influences GLP-1 secretion through the IL-6/STAT3/Akt/GSK3β/β-catenin signaling pathway.

## Discussion

This study suggests two main conclusions. First, analytical evidence from the cellular level to clinical samples demonstrates a negative correlation between PANDER and GLP-1. Secondly, PANDER inhibits GLP-1 expression and secretion through the IL-6/JAK/STAT3/PI3K/Akt pathway, suggesting the presence of regulatory pathways among pancreatic factors, inflammatory factors, and gut hormones in glucose metabolism, providing a novel perspective for the prevention and treatment of diabetes.

Postpartum patients with a history of GDM history were chosen to examine the correlation between serum levels of GLP-1 and PANDER, rather than patients with T2DM in the present study. Evidence has shown that the PANDER levels are significantly elevated in patients with gestational diabetes, MS, and T2DM patients (11, 13, 15). Thus, we believe that the effect of PANDER on glucose metabolism is consistent among these populations. We performed glucose tolerance screening for women with GDM at 4–12 weeks postpartum, following the ACOG 2018 guideline recommendations (25). Combined with our latest evidence, suggesting that abnormal elevation of PANDER indicates the risk of postpartum progression to T2DM in GDM patients (20), this study further elaborates on the significance of the influence of PANDER on glucose metabolism in the T2DM population, indicating that high levels of PANDER may contribute to GDM history being a high-risk factor for T2DM.

Unexpectedly, in contrast with previous results* in vitro*, IL-6 was positively correlated with PANDER and negatively correlated with GLP-1. There could be several reasons contributing to the discrepancy. First, this discrepancy was likely due to a species difference between humans and mice (26). In support of this point, the Ellingsgaard group identified IL-6 as an important determinant for the stimulation of GLP-1 secretion from mouse enteroendocrine cells *in vitro* (23). The same group further investigated the effect of IL-6 administration on GLP-1 and post-prandial glucose excursion in humans. The finding was inconsistent with previous rodent work. They found that, with increased peripheral circulating IL-6 levels, GLP-1 levels did not change. Surprisingly, IL-6 improved glucose tolerance in a GLP-1-independent pathway but decreased insulin secretion in a GLP-1-dependent pathway, which remains unexplained (24). Secondly, because IL-6 was also secreted from skeletal muscle cells and adipose tissue (27), BMI of participants and exercise may be key point to the difference. In contrast with our results, obese participants (BMI ≥30 and ≤40 kg/m^2^) from a randomized, placebo-controlled, double-blind, multi-center study had active GLP-1 levels that were 26% lower with IL-6 receptor antagonist (tocilizumab) compared with placebo during the acute exercise bout (24). Lastly, subjects in the current study were in a special physiological state due to lactation. The hormone levels of women during lactation changed significantly. The relationship between these variations and the role of IL-6 in glucose metabolism was unknown. These results have been added to the research findings.

Our previous research revealed that PANDER was discovered to present a similar biodistribution in the liver, pancreas, small intestine, and bone in mice through F-18 radiotracer labeling, indicating that the intestine is one of the sources of PANDER biosynthesis (28). The expression of GCG and secretion of GLP-1 in the intestinal L-cell line GLUTag cells were inhibited when treated with exogenous PANDER, while serum GLP-1 levels were decreased in mice overexpressing PANDER (16). Combined with the previous research conducted by our research group, the present study further validates the correlation between PANDER and GLP-1 in clinical research. A negative correlation between serum PANDER levels and GLP-1 levels in postpartum women with a history of GDM was observed, which is consistent with the phenomenon that PANDER may inhibit GLP-1 secretion in both *in vivo* and *in vitro* studies (16). Therefore, through experiments conducted at multiple levels, including cellular, animal, and clinical studies, we have confirmed that PANDER influences the secretion of the gut hormone GLP-1. These findings suggest the existence of a pancreatic–intestinal cross-talk pathway that regulates glucose metabolism.

STC-1 cells, as a mouse-derived model of intestinal endocrine cells, provide a good molecular research foundation for studying GLP-1 secretion (29). Subsequently, we used STC-1 cells as a research tool to investigate the impact of PANDER on GLP-1 secretion. Genome-wide transcriptome profiling of PANDER-overexpressing STC-1 cells showed that IL-6 and its related signaling pathway presented significant variability in response to PANDER overexpression. Combined with existing literature evidence, we validated the changes in the expression levels of IL-6 and its related signaling pathways in both PANDER overexpressing and knockdown STC-1 cells. The results revealed a negative correlation between PANDER and IL-6 levels and the related STAT3/Akt/GSK3β/β-catenin signaling pathway. Rescue experiments in both PANDER-overexpressing and knockdown STC-1 cells further validated the mediating effect of IL-6 and its related signaling pathways on the negative regulatory effect of PANDER on GLP-1.

Evidences have shown that the regulation of glucose metabolism by IL-6 has a dual-edged effect. Previously, elevated circulating levels of IL-6 were considered an independent predictive factor for T2DM and were associated with the development of inflammation, insulin resistance, and β-cell dysfunction (30). However, recent evidence of clinical and experimental studies has demonstrated the promoting effects of IL-6 on GLP-1 secretion and synthesis, which may improve glucose metabolism. Ellingsgaard *et al*. recently reported that tocilizumab, an IL-6 receptor antagonist, inhibited the increase of GLP-1 secretion (24). Moreover, IL-6 activates GLP-1 secretion through the upregulation of the Pcsk1 gene, Slc5a1 gene, and Slc2a1 gene, which leads to increased GCG gene expression and glucose intake (23). In addition, IL-6 may also modulate the secretion of GLP-1 by promoting the release of leptin from adipocytes (31), activating GCG gene expression in pancreatic α cells (32), and mediating the effect of endotoxin (33) and GIP (34) on pancreatic α cells. Combining the results of the gene chip in the present study, we subsequently performed reversal experiments by treating the PANDER-overexpressing group with exogenous IL-6, treating the PANDER-knockdown group with a specific inhibitor of the JAK/STAT3 signaling pathway (AG490), and establishing the PANDER-IL-6 double-knockdown STC-1 cells, which successfully reversed the effect of PANDER on GLP-1 secretion. Therefore, the present study confirmed that IL-6 indeed mediates the regulatory effect of PANDER on GLP-1.

It has been reported that the regulation mechanism of IL-6 on GLP-1 relates to the activation of the JAK/STAT3 pathway, which starts with gp130 phosphorylation (30). Activation of JAK further activates the RAF/MEK/ERK (MAPK) pathway and the PI3K/AKT pathway (35). Then the downstream WNT signaling pathway promotes GCG gene expression and GLP-1 secretion through β-catenin and TCF7L2 translocation into the nucleus and binding to the G2 enhancer module of the GCG gene (36). Previous studies have demonstrated that bone marrow-derived growth factors, insulin, and lithium carbonate affect GLP-1 secretion in intestinal L cells through the WNT/β-cat signaling pathway (37, 38, 39). Thus, we further explored the regulatory effect of endogenous PANDER on the STAT3/Akt/GSK3β/β-cat signaling pathway. PANDER overexpression inhibited the STAT3/Akt/GSK3β/β-catenin signaling pathway, while knockdown of PANDER had the opposite effect. Further rescue experiments with IL-6 and AG490 confirmed these negative regulatory effects. Overall, these findings suggest that regulating endogenous PANDER expression through the IL-6/STAT3/Akt/GSK3β/β-catenin signaling pathway is critical for impacting GLP-1 secretion in STC-1 cells.

The relationship between PANDER and GLP-1 suggests the existence of the intestinal feedback of pancreatic islet function regulation. Chen *et al.* (40) found that GLP-1 may antagonize the trigger effect of free fatty acids in the expression of PANDER and inhibit PANDER-related apoptosis in pancreatic islet β cells. And it has been widely confirmed that in patients with diabetes, after receiving GLP-1 receptor agonist treatment, the level of IL-6 decreases and pancreatic islet function improves (41, 42), suggesting that GLP-1 may affect the function of pancreatic islets through the negative feedback of IL-6. Treatment with rilalutide in STZ-induced diabetic rats inhibits the level of IL-6 in a time-dependent manner and improves the apoptosis in pancreatic islets (43). Evidence that GLP-1 stimulates the expression of specific inflammatory markers, including IL-6, have been observed in db/db mice (44), Wistar diabetic rats (45), ischemia-reperfusion injury model (46) in mice, and septic mouse models (47). The above evidence has fully demonstrated that the gut hormone GLP-1 can inhibit the systemic inflammatory response and regulate the function of pancreatic islets based on this pancreas–inflammation–intestine regulatory axis, which once again clarifies the inseparable relationship between the pancreas, inflammation, and intestine in glucose metabolism. The PANDER–IL-6–GLP-1 regulatory mechanism highlights the crucial role of the pancreas–inflammation–intestine axis in the regulation of glucose metabolism. Despite its significance, the physiological mechanism of PANDER remains largely unexplored. Fangfang *et al.* (48) identified the fibroblast growth factor receptor to be the specific binding target of PANDER in Xenopus. However, it has not been verified in other species. The results of the present study provide the first evidence of the mediating role of IL-6 in the physiological mechanism related to PANDER, further supporting the importance of the pancreas-inflammation-intestine axis in glucose regulation.

There are still some limitations to this study. First, although there is *in vitro* experimental data on the impact of endogenous PANDER and IL-6 expression on GLP-1, further *in vivo* experiments are required to confirm the regulatory mechanism of the PANDER–IL-6–GLP-1 axis. Secondly, the activation of apoptosis-related pathways in STC-1 cells overexpressing PANDER, as suggested by RNA-seq results, requires further exploration to understand the role of apoptosis in mediating the influence of PANDER on GLP-1 secretion. Thirdly, there is currently no evidence of PANDER expression in the pathological state of human intestinal endocrine cells. Our cell model construction results indicate that PANDER levels were overexpressed by more than thousands of folds (5000×–10000×), which is likely outside the physiological range, and the results should be interpreted with caution. Lastly, the specific binding target through which PANDER affects IL-6 has yet to be identified. Therefore, additional studies are needed to fully comprehend the intricate interplay between PANDER and IL-6 in regulating GLP-1 secretion. Identifying this binding target could offer valuable insights into the molecular mechanisms underlying GLP-1 regulation and potential therapeutic targets for diabetes management.

## Conclusion

**Figure 5:**
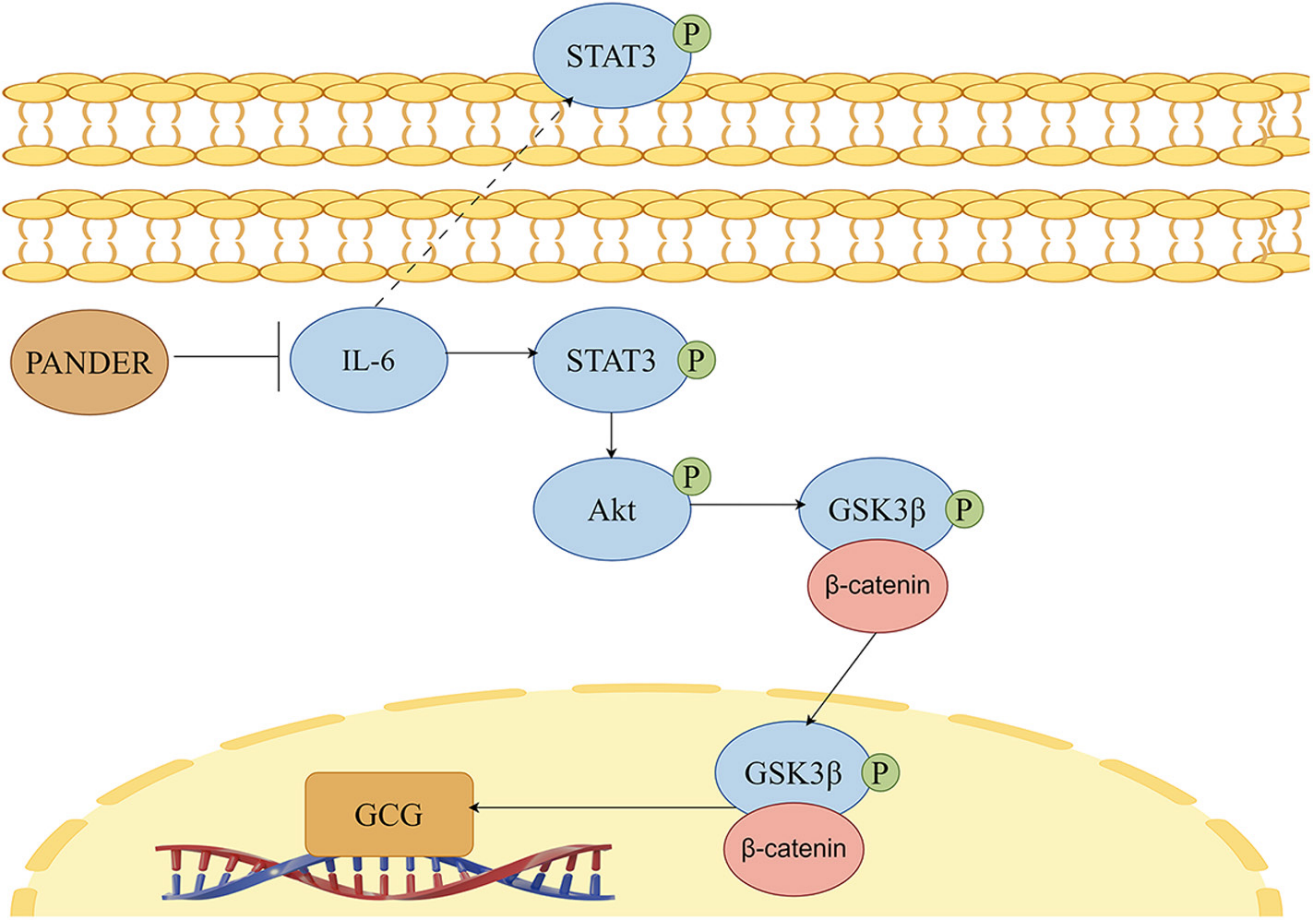
PANDER regulates GLP-1 through the IL-6-related STAT3/Akt/GSK3β/β-catenin signaling pathway in STC-1 cells.

This study is the first to investigate impaired GLP-1 secretion in GDM patients during the early postpartum period and its association with elevated PANDER levels. Our findings demonstrate the inhibitory effect of PANDER on GLP-1 secretion *in vitro.* Moreover, we have discovered a novel regulatory role of PANDER in intestinal endocrine cells by modulating IL-6 and its downstream signaling pathways. Furthermore, our experiments confirmed that IL-6 and its downstream signaling pathways mediate the regulation of PANDER on GLP-1. This interaction mechanism between PANDER and IL-6 may be a key pathophysiological mechanism for PANDER in regulating glucose metabolism. These results expand upon the theoretical basis for the closed-loop feedback regulation mechanism of the pancreas–inflammation–intestine regulatory axis in glucose metabolism (Fig. 5).

## Supplementary Materials

Supplementary Figure 1

Supplementary Figure 4

Supplementary Tables

Supplementary Data

## Declaration of interest

The authors declare that they have no known competing financial interests or personal relationships that could have appeared to influence the work reported in this paper.

## Funding

This study was supported by grants from the Clinical Medical 5010 Project Foundation of Sun Yat-sen Universityhttp://dx.doi.org/10.13039/501100002402 (Grant number: 2017001), the Science and Technology Projects in Guangzhou (Grant number: 2024A04J4096), the Fundamental Research Funds for the Central Universitieshttp://dx.doi.org/10.13039/501100012226 (Grant number: 21623306), and Administration of Traditional Chinese Medicine of Guangdong Province, China (Grant number: 20241069).

## Patient consent

Written consent has been obtained from each patient or subject after a full explanation of the purpose and nature of all procedures used.

## Author contribution statement

Li Zeting is responsible for article writing, cell line construction, and functional identification. Pei Ling is responsible for clinical sample collection and statistics. Xiao Huangmeng is responsible for RNA chip differential gene analysis. Chen Nan, Lai Fenghua, Yue Shufang, and Xu Changliu are responsible for discussing experimental methods. Li Yanbing and Xiao Haipeng are responsible for guiding research ideas. Cao Xiaopei is responsible for formulating research plans, as well as polishing and revising the article.
