# Supplementary figures and images for: The role of PANDER and its interplay with IL-6 in the regulation of GLP-1 secretion
Source: Endocr Connect. 2024 Oct 4;13(11):e230548. doi: 10.1530/EC-23-0548 (PMC11466252; doi:10.1530/EC-23-0548)

A

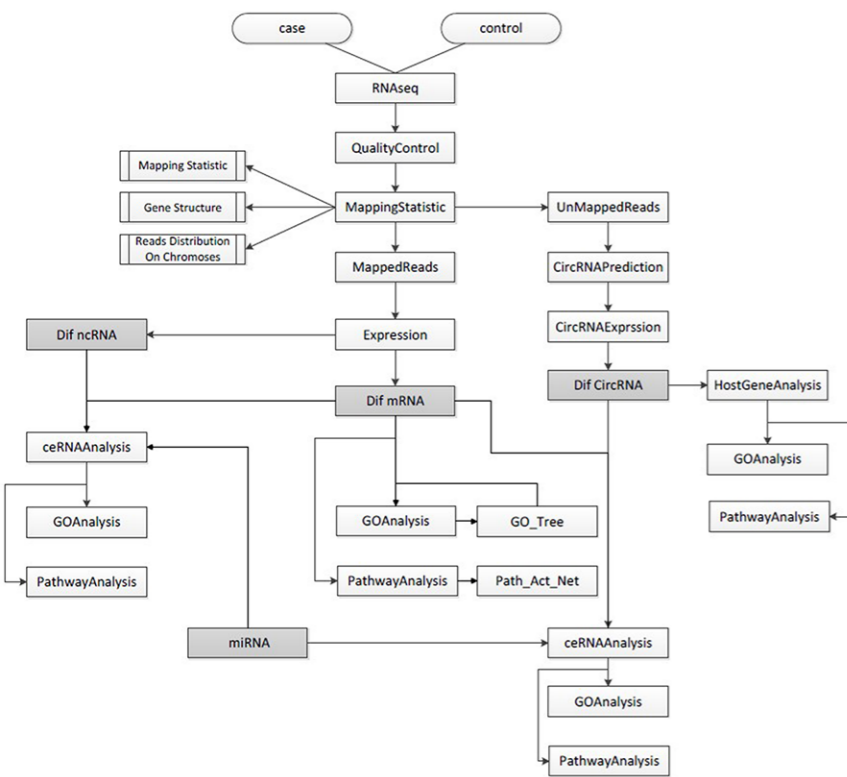

B

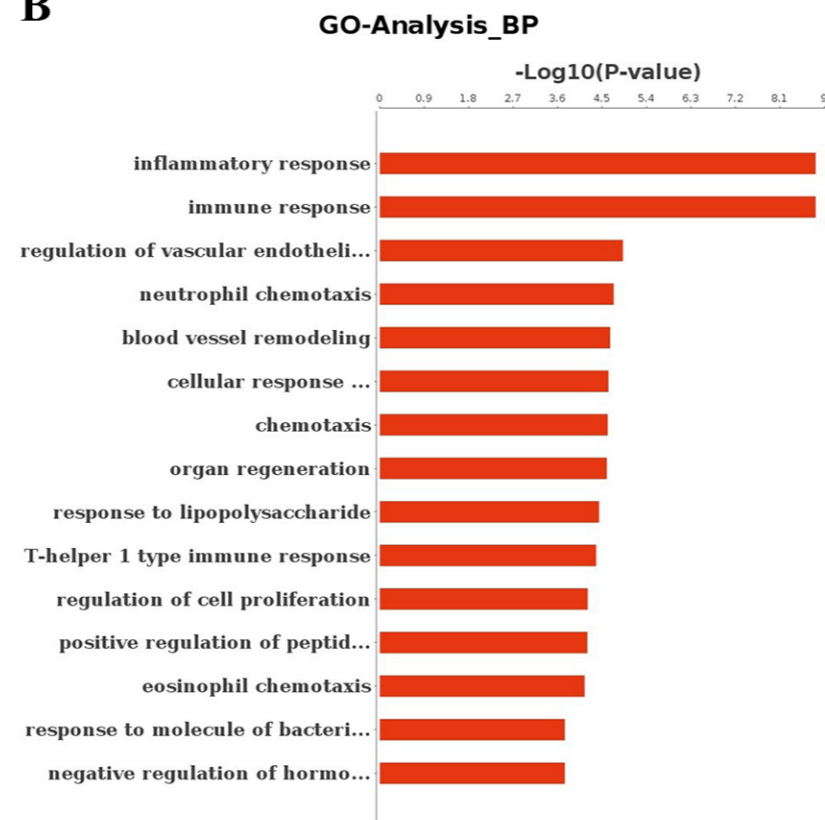

C

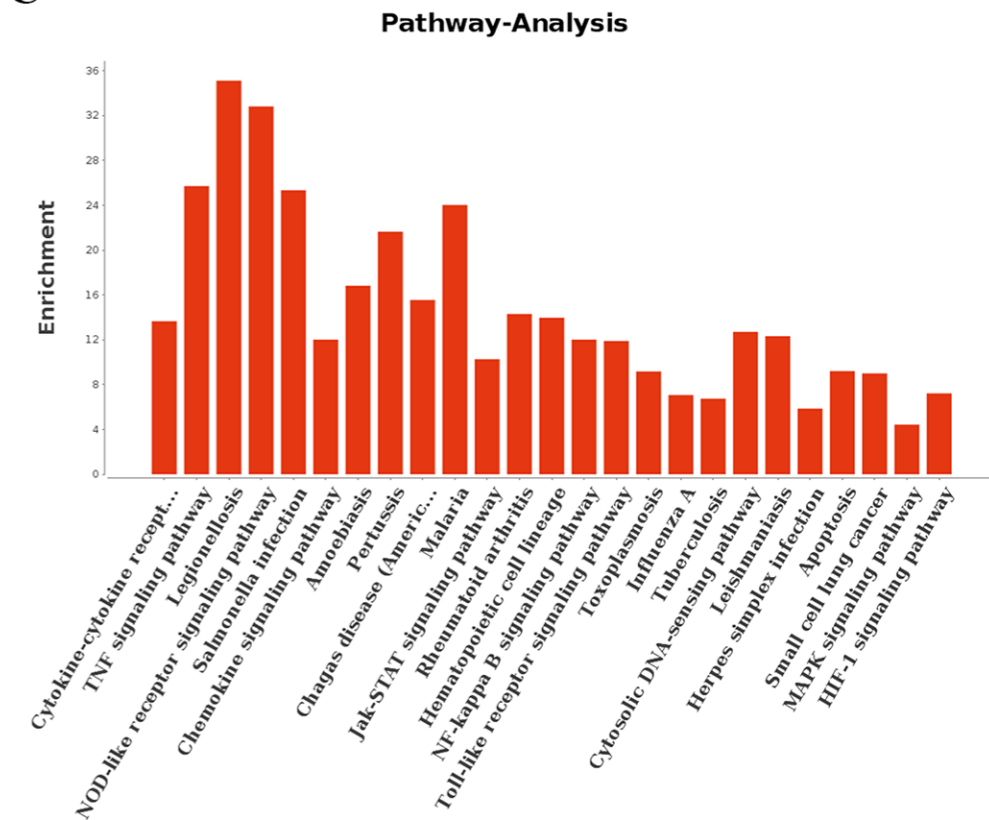

D

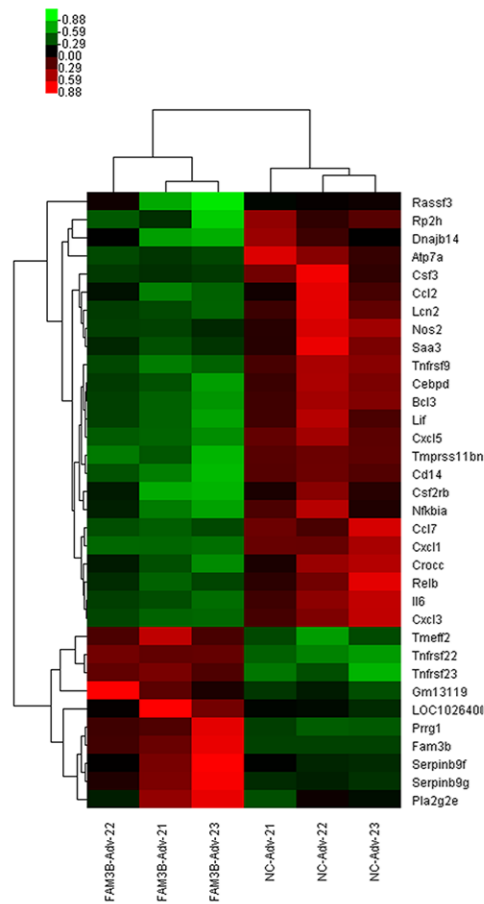

E

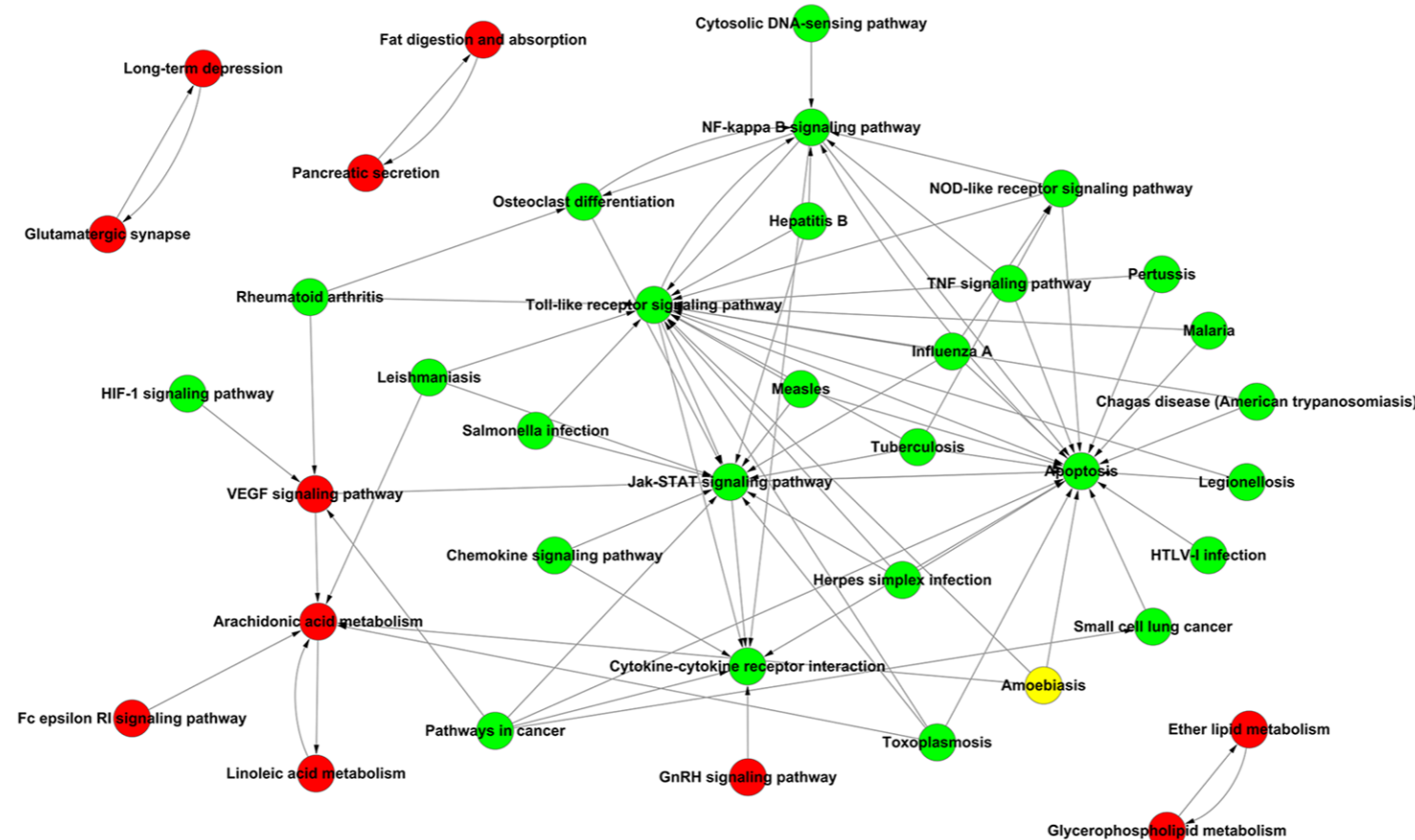

F

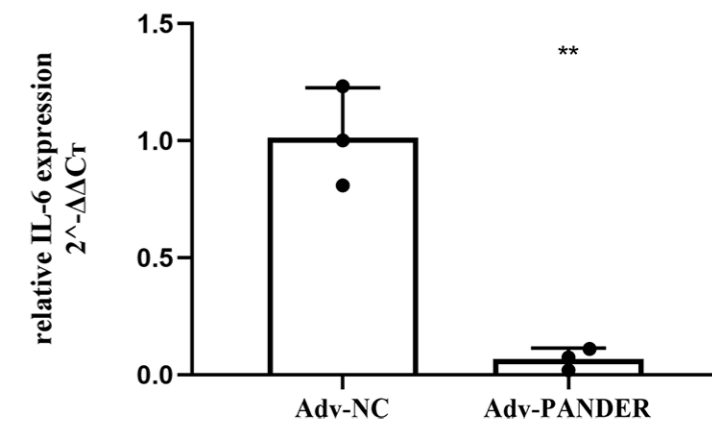

Supplement: Supplementary Figure 1 [file supplementary_figure_1.pdf]

Figure 4 A-E

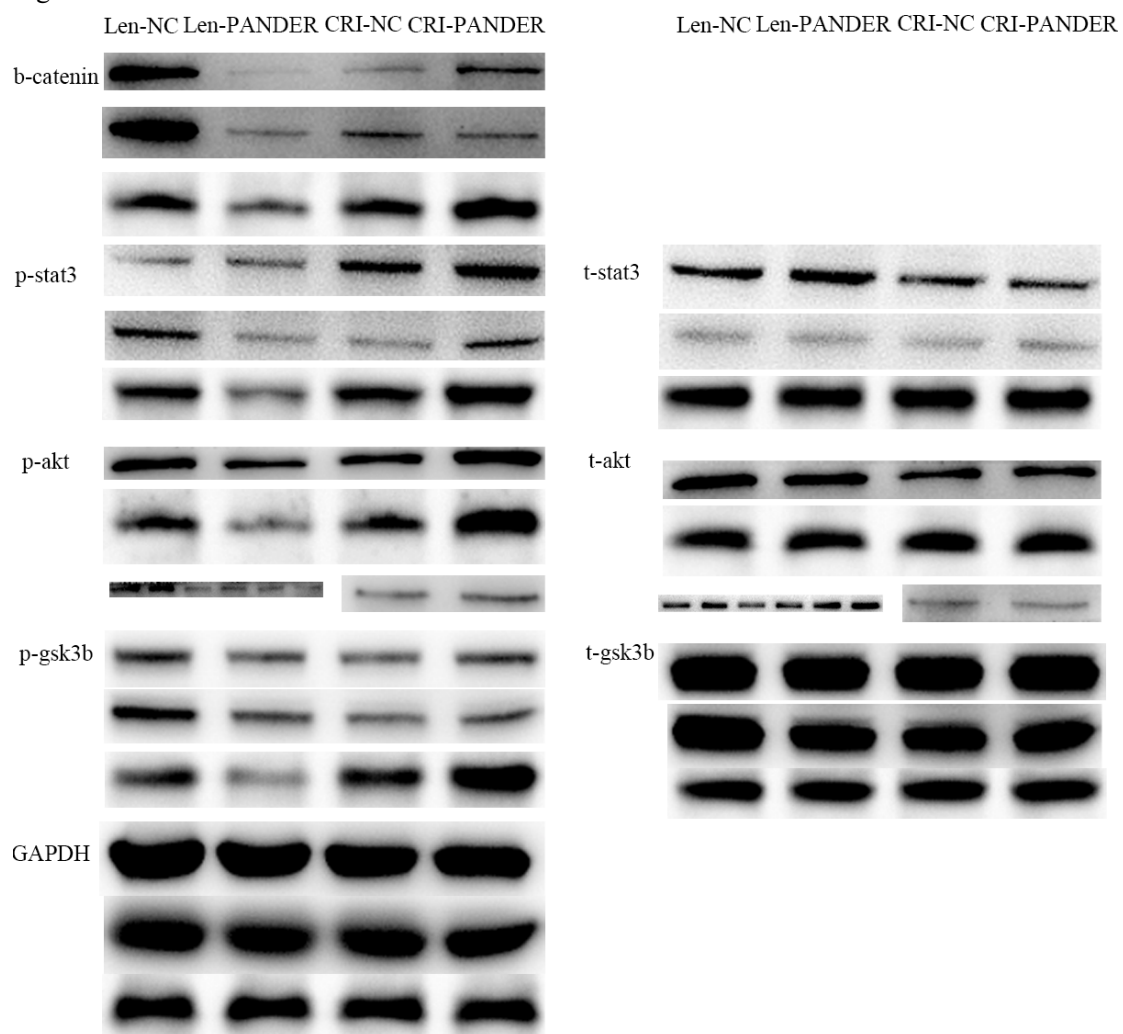

Figure 4 F-J

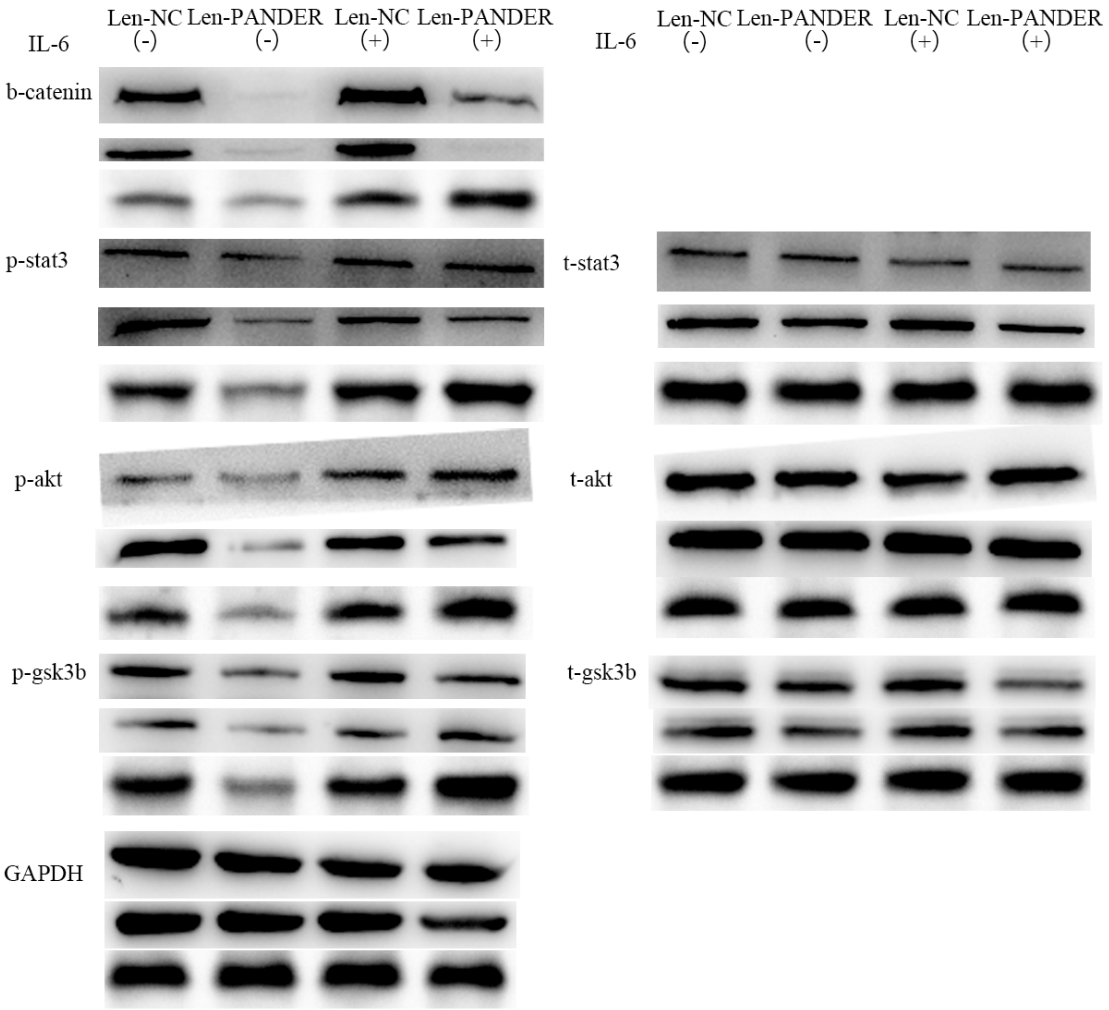

Figure 4 K-O

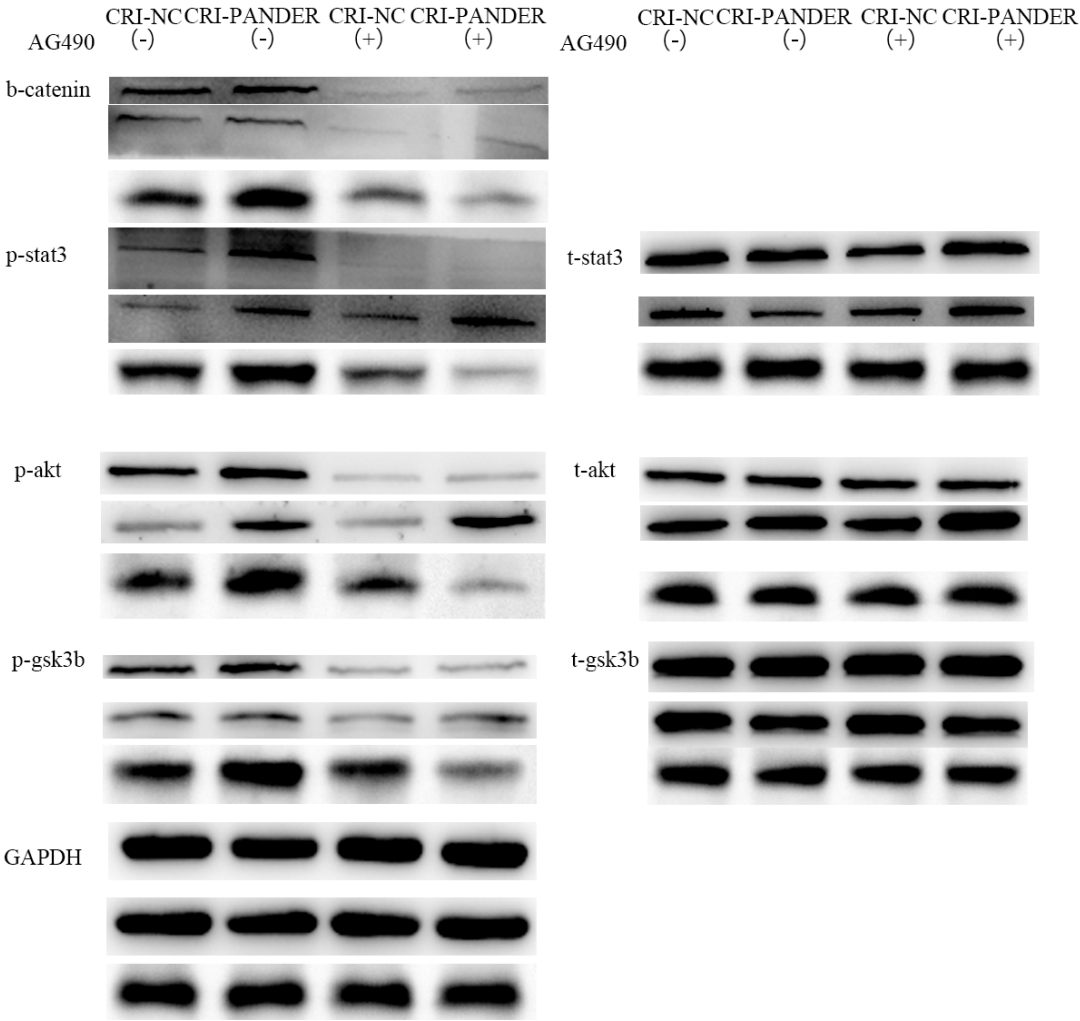

Figure 1 I, N & 4P, R

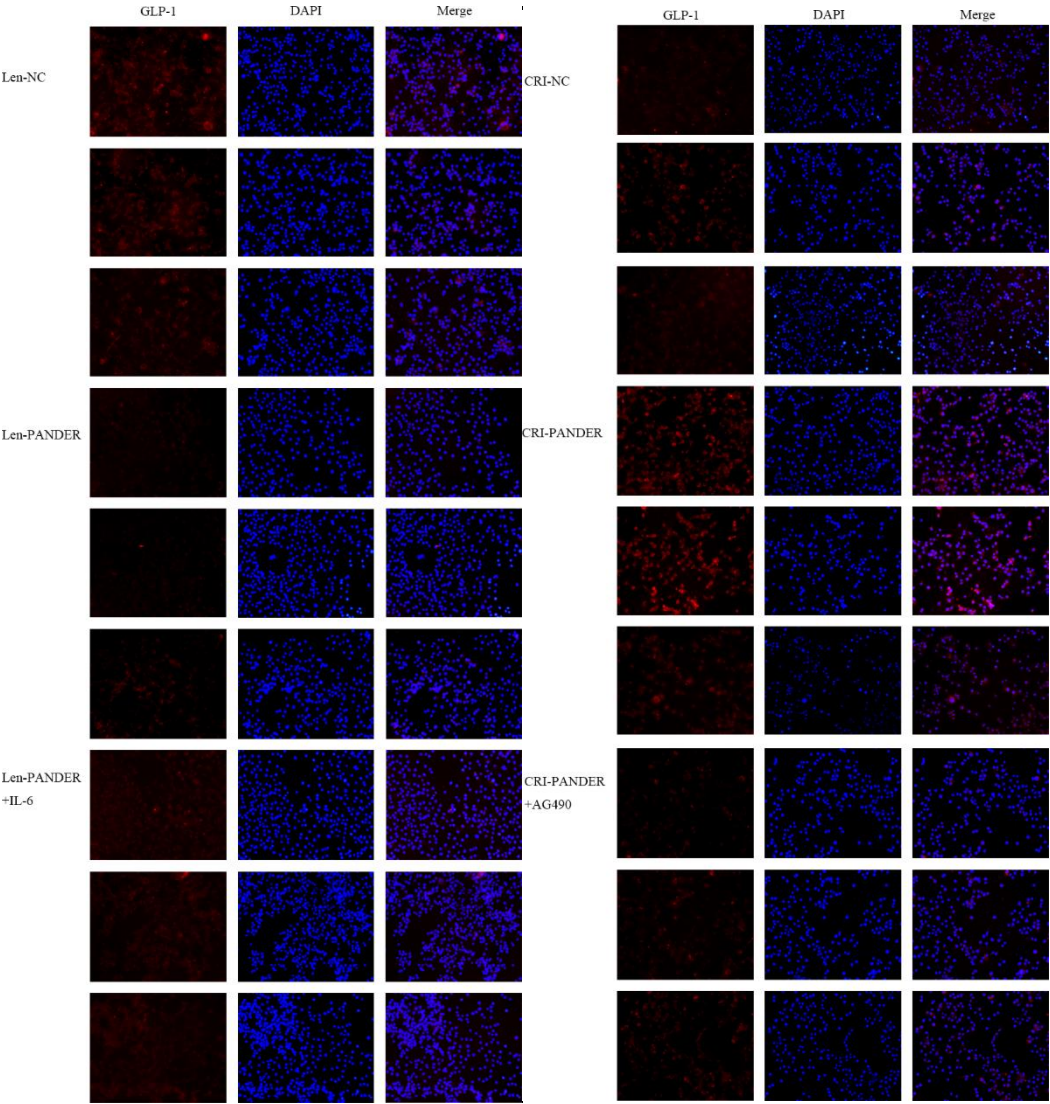

Supplement: Supplementary Figure 4 [file supplementary_figure_4.pdf]
