# Supplementary material for: The role of PANDER and its interplay with IL-6 in the regulation of GLP-1 secretion
Source: Endocr Connect. 2024 Oct 4;13(11):e230548. doi: 10.1530/EC-23-0548 (PMC11466252; doi:10.1530/EC-23-0548)
Supplement: Supplementary Tables [file supplementary_tables.pdf]

Supplementary table 1 RT-PCR primer sequence

Primer	**Sequence**
β-actin Forward	5'-GATCATTGCTCCTCCTGAGC-3'
β-actin Reverse	5'-ACTCCTGCTTGCTGATCCAC-3'
PANDER Forward	5'-TCTGGAAGATGCGTCCAGTTG-3'
PANDER Reverse	5'-TGTAGAGAGTGCTGGACAGGG-3'
GCG Forward	5'-CGCCACTCACAGGGCACATT-3'
GCG Reverse	5'-CAAAATCTTGGGCACGGCGG-3'
IL-6 Forward	5'-TTCCAATGCTCTCCTAACAGAT-3'
IL-6 Reverse	5'-ATGAATTGGATGGTCTTGGTCC-3'

Supplementary table 2 PANDER cDNA sequence

5'-CCCTGTGCGCCTGGTATTCTGGGTACCTGCTCGCGGAGCTCATTCCTG
ACGTGCCCCTGTCCAGCACTCTCTACAACATCCGAAGCATTGGAGAGAG
ACCTGTTCTCAAAGCCCCAGCCCCCAAAAGACAAAAATGTGACCATTGG
TCCCCATGTCCTCCTGACACCTATGCCTACCGGCTGCTCAGTGGTGGTGG
CCGCGACAAGTATGCCAAGATCTGCTTTGAGGATGAAGTGCTAATAGGA
GAGAAGACGGGGAATGTGGCAAGAGGGATAAACATTGCTGTCGTCAAC
TATGAGACAGGAAAAGTGATAGCGACAAAGTACTTTGATATGTATGAA
GGTGATAACTCCGGGCCAATGGCCAAGTTCATTCAGAGCACTCCTTCAA
AATCCCTGCTGTTCATGGTGACTCATGATGATGGAAGTTCCAAACTGAA
GGCTCAAGCAAAGGATGCCATAGAAGCCCTTGGAAGCAAAGAAATCAA
GAACATGAAGTTCAGATCAAGCTGGGTGTTTGTTGCAGCAAAGGGCTTT
GAGCTCCCTTCAGAAATCGAGAGAGAAAAAATCAACCACTCAGATCAA
TCCAGGAACAGATATGCAGGCTGGCCAGCGGAGATCCAGATCGAAGGA
TGCATACCCAAAGGGCTGAGATAA-3'

Supplementary table 3 CRISPR-sgRNA primer sequence

primer	**Sequence**
PANDER oligo 1	5'-CACCGCCTTCGATCTGGATCTCCGC-3'
PANDER oligo 2	5'-AAACGCGGAGATCCAGATCGAAGGC-3'
